# *Streptococcus pyogenes* Phospholipase A_2_ Induces the Expression of Adhesion Molecules on Human Umbilical Vein Endothelial Cells and Aorta of Mice

**DOI:** 10.3389/fcimb.2017.00300

**Published:** 2017-06-30

**Authors:** Masataka Oda, Hisanori Domon, Mie Kurosawa, Toshihito Isono, Tomoki Maekawa, Masaya Yamaguchi, Shigetada Kawabata, Yutaka Terao

**Affiliations:** ^1^Division of Microbiology and Infectious Diseases, Graduate School of Medical and Dental Sciences, Niigata UniversityNiigata, Japan; ^2^Department of Microbiology and Infection Control Sciences, Kyoto Pharmaceutical UniversityKyoto, Japan; ^3^Research Center for Advanced Oral Science, Graduate School of Medical and Dental Sciences, Niigata UniversityNiigata, Japan; ^4^Department of Oral and Molecular Microbiology, Osaka University, Graduate School of DentistryOsaka, Japan

**Keywords:** *S. pyogenes*, phospholipase A_2_, SlaA, HUVEC, ICAM1, VCAM1

## Abstract

The *Streptococcus pyogenes* phospholipase A_2_ (SlaA) gene is highly conserved in the M3 serotype of group A *S. pyogenes*, which often involves hypervirulent clones. However, the role of SlaA in *S. pyogenes* pathogenesis is unclear. Herein, we report that SlaA induces the expression of intercellular adhesion molecule 1 (ICAM1) and vascular cell adhesion molecule 1 (VCAM1) via the arachidonic acid signaling cascade. Notably, recombinant SlaA induced ICAM1 and VCAM1 expression in human umbilical vein endothelial cells (HUVECs), resulting in enhanced adhesion of human monocytic leukemia (THP-1) cells. However, C134A, a variant enzyme with no enzymatic activity, did not induce such events. In addition, culture supernatants from *S. pyogenes* SSI-1 enhanced the adhesion of THP-1 cells to HUVECs, but culture supernatants from the Δ*slaA* isogenic mutant strain had limited effects. Aspirin, a cyclooxygenase 2 inhibitor, prevented the adhesion of THP-1 cells to HUVECs and did not induce ICAM1 and VCAM1 expression in HUVECs treated with SlaA. However, zileuton, a 5-lipoxygenase inhibitor, did not exhibit such effects. Furthermore, pre-administration of aspirin in mice intravenously injected with SlaA attenuated the transcriptional abundance of ICAM1 and VCAM1 in the aorta. These results suggested that SlaA from *S. pyogenes* stimulates the expression of adhesion molecules in vascular endothelial cells. Thus, SlaA contributes to the inflammation of vascular endothelial cells upon *S. pyogenes* infection.

## Introduction

*Streptococcus pyogenes* is a gram-positive bacterium that can cause superficial infections, such as pharyngitis and pyoderma; invasive infections, such as necrotizing fasciitis and streptococcal toxic shock syndrome; and post-infectious diseases, such as rheumatic fever (Cunningham, 2000). *S. pyogenes* produces many extracellular molecules that contribute to host–pathogen interactions (Musser and Krause, 1998; Cunningham, 2000; Fischetti, 2000; Kurosawa et al., 2016) and most of the extracellular molecules possess enzymatic activities against host tissues and the immune system (Terao et al., 2006, 2008; Honda-Ogawa et al., 2013). Epidemiological studies have shown that M3 serotype strains are the second most common cause of invasive infections in the United States, Canada, Western Europe, Japan, and Israel (Murakami et al., 2002; Terao et al., 2002; Li et al., 2003; Moses et al., 2003; Muller et al., 2003; Nakagawa et al., 2003; Schmitz et al., 2003). Surveillance studies have also revealed that M3 strains cause a higher rate of severe invasive diseases such as necrotizing fasciitis and death than other M-type strains (O'Brien et al., 2002; Sharkawy et al., 2002).

*S. pyogenes* is a diverse species with strain-specific virulence genes derived from multiple prophages (Banks et al., 2002; Beres and Musser, 2007). Genome sequencing analysis of *S. pyogenes* M1–M6, M12, M18, M28, and M49 serotype strains indicated that these strains have 4–8 prophages or prophage-like elements (Nozawa et al., 2011). We identified that M3 serotype strain SSI-1 possesses the phospholipase A_2_ (PLA_2_) gene, designated as *slaA*, on the prophage genome. Beres et al. (2004) reported that the enhanced capacity for invasive infection incurred by M3 correlated with the acquisition of prophage genomes encoding the *slaA* gene. SlaA has a conserved region of amino acid residues found in several secreted PLA_2_ enzymes, such as those found in snake venom (Pearson et al., 1993).

Sitkiewicz et al. (2007) reported that SlaA enhanced the binding of *S. pyogenes* to human epithelial cells, such as immortalized pharyngeal cells and human tracheobronchial epithelial cells. However, the pathogenesis due to SlaA in host cells remains unclear. Yokote et al. (1993) reported that endogenous PLA_2_ from human endothelial cells induces the expression of intercellular adhesion molecule 1 (ICAM1) and vascular cell adhesion molecule 1 (VCAM1). In addition, Zhu et al. (1999) reported that cytosolic PLA_2_ in human eosinophils stimulated the expression of β1- and β2-integrin, resulting in binding to ICAM1 and VCAM1. In this study, we hypothesized that SlaA induces the expression of adhesion molecules. To confirm this hypothesis, we examined the adhesion of human monocytic leukemia cell line (THP-1) cells to human umbilical vein endothelial cells (HUVECs) and the association of this with the expression of adhesion molecules, such as ICAM1, VCAM1, E-selectin, and P-selectin, on HUVECs treated with SlaA.

## Materials and methods

### Reagents and animals

Aspirin and zileuton were purchased from Merck. Aspirin was solubilized in phosphate-buffered saline (PBS) containing 0.5% ethanol (*in vitro*) or 0.5% CMC (*in vivo*). All other drugs were of analytical grade. Male, 10–12-week-old BALB/c mice (Nihon CLEA, Japan) were used in this study. Mice were maintained under standard conditions in accordance with our institutional guidelines. All animal experiments were approved by the Institutional Animal Care and Use Committee of Niigata University.

### Bacterial strains

*S. pyogenes* clinical strains were generously provided by Dr. Kawabata of Osaka University (Table 1) (Murakami et al., 2002; Terao et al., 2002). All strains were grown in Todd Hewitt broth (Becton Dickinson, MD, USA) supplemented with 0.2% yeast extract (THY broth) at 37°C.

**Table 1:** Expression of SlaA in various *S. pyogenes* strains.

**Strain name**	**M serotype**	**Reference**	***slaA* (gene)**	**SlaA (protein)**
NIH11	1	Invasive	−	−
NIH17	1	Invasive	−	−
NIH22	1	Invasive	−	−
NIH27	1	Invasive	−	−
TW3354	1	Non-invasive	−	−
SSI-1	3	Invasive	+	+
SSI-35	3	Invasive	+	+
NIH1	3	Invasive	+	+
NIH16	3	Invasive	+	+
NIH21	3	Invasive	+	+
TW3358	3	Non-invasive	+	+
NIH2	4	Invasive	−	−
NIH6	4	Invasive	−	−
TW3392	4	Non-invasive	+	+
TW3398	4	Non-invasive	+	+
TW3400	4	Non-invasive	+	+
TW3341	11	Non-invasive	−	−
TW3360	11	Non-invasive	−	−
TW3337	12	Non-invasive	−	−
TW3344	12	Non-invasive	−	−
NIH35	28	Invasive	−	−
TW3357	28	Non-invasive	−	−
TW3374	75	Non-invasive	−	−
TW3364	75	Non-invasive	−	−
TW3365	75	Non-invasive	−	−
TW3419	89	Non-invasive	−	−
TW3551	89	Non-invasive	−	−

*+, Detected; −, not detected*.

### Detection of *slaA* gene

Chromosomal DNA from *S. pyogenes* was purified with Bactozol (Molecular Research Center, Inc., Cincinnati, OH, USA). The forward primer (5′-GGGGGATCCGGGATAAATGATAAAATGGAA-3′) and reverse primer (5′-CCCGAATTCTTAACATCCTATAGAACCTAC-3′) were used to amplify the *slaA* gene of various *S. pyogenes* strains.

### Integration mutagenesis

The PCR product of the internal portion of the *slaA* gene was amplified using forward (5′-GGGAAGCTTATGAAAAAAGTAATAAATACTATTCTATAAGCTGCT-3′) and reverse (5′-CCCGGATCCTTAACATCCTATAGAACCTACTGTCTCAAAATATAC-3′) primers and ligated into a thermosensitive suicide vector pSET4s (Takamatsu et al., 2001a,b). pSET4s was kindly provided by Dr. Takamatsu (The National Agriculture and Food Research Organization, Japan). The constructed plasmid was transformed into wild-type strain SST-1 by electroporation (2.5 kV, 25 μF, and 600 Ω), and the inactivated mutant strain was selected on spectinomycin-containing agar plates.

### Detection of SlaA protein

*S. pyogenes* strains were incubated in THY broth at 37°C for 6 h to an OD_610_ of 0.3–0.4. The bacteria were collected, washed in PBS, and suspended in 1 ml of PBS. Bacteria (100 μl) were added to the HUVECs (2 ml, 6-well plate) and incubated at 37°C for 5 h. Culture medium was centrifuged at 3,000 × g for 15 min, and the culture supernatants were concentrated to 100 μl with Amicon Ultra Centrifugal Filters 10K (Merck Millipore, Billerica, MA, USA). The samples were mixed with 2% SDS-sample buffer containing 1% 2-mercaptoethanol, boiled for 3 min, then separated by SDS-PAGE using 12.5% gels, and transferred to polyvinylidene difluoride membranes (Merck Millipore). The membrane was blocked and incubated with an anti-SlaA antibody and HRP-conjugated secondary antibody. The membrane was treated with HRP substrates and analyzed using a chemiluminescence detector (Fujifilm, Tokyo, Japan). The anti-SlaA antibody was produced in rabbits intramuscularly administered SlaA protein (Eurofin, Tokyo, Japan).

### Construction of recombinant SlaA

A recombinant (r) SlaA expression plasmid was constructed using a pGEX-6P-1 vector (GE Healthcare, Uppsala, Sweden). The forward primer (5′-GGGGGATCCGGGATAAATGATAAAATGGAA-3′) and reverse primer (5′-CCCGAATTCTTAACATCCTATAGAACCTAC-3′) were used to amplify the *slaA* gene of *S. pyogenes* strain SSI-1 by PCR. The resultant PCR fragment was cloned into a pGEX-6P-1 vector. The pGEX-6P-1 vector containing the *slaA* gene was transformed into *Escherichia coli* strain DH5α (TaKaRa, Shiga, Japan) by the heat shock method. The DH5α transformants were grown in Luria-Bertani broth (Nacalai Tesque, Kyoto, Japan) supplemented with 100 μg/ml ampicillin (Meiji Seika, Tokyo, Japan) to select for the pGEX-6P-1 vector. Then, the rSlaA protein was purified using glutathione-Sepharose 4B beads (GE Healthcare), and the GST tag was cleaved by PreScission Protease (GE Healthcare). The purified rSlaA protein was dialyzed against PBS. The amount of lipopolysaccharide (LPS) in 1 μg of purified rSlaA protein was determined to be less than 2 pg using the LPS detection kit (GenScript, Piscataway, NJ, USA).

### Site-directed mutagenesis

The KOD-plus Mutagenesis kit (TOYOBO, Osaka, Japan) was used with the following primers to the modified plasmid (pGEX6P1-SlaA): C134A, 5′-GCCCAAAACCACGATAGTTGCTATAAGTGG-3′ and 5′-ACCTTGATCCAAAACATCTACTACTGGCAA-3′. The purified C134A were assayed for phospholipase A_2_ activity with a phospholipase A_2_ assay kit (Cayman Chemical, Ann Arbor, MI, USA). In line with a previous report (Nagiec et al., 2004), the enzymatic activity of C134A was not detected.

### Phospholipase A2 activity

Phospholipase A2 activity of the recombinant protein (rSlaA and C134A) and culture supernatant of SSI-1 and Δ*slaA* isogenic mutant strain was measured by Phospholipase A2 colorimetric assay kit (Cayman, Michigan, USA).

### Human monocytic cells

Human THP-1 monocytes were purchased from RIKEN (Tsukuba, Japan). These cells were cultured in RPMI-1640 medium supplemented with 10% (v/v) fetal bovine serum, 100 U/ml penicillin, 100 μg/ml streptomycin, and 2 mM L-glutamine. The culture was maintained at 37°C in 5% CO_2_. The cells were stained with 5-(and 6)-carboxyfluorescein diacetate succinimidyl ester (CFSE; Thermo Fisher Scientific, Eugene, OR, USA) at 37°C for 60 min, and then washed.

### HUVECs

HUVECs were purchased from Lonza Inc. They were cultured to confluence in EGM-2 medium at 37°C in 5% CO_2_. HUVECs were used within 24 h after reaching confluence, between passages 3 and 5.

### Assay for monocyte adhesion to endothelial cells

After the stimulation of HUVECs with SlaA, C134A, or various culture supernatants from *S. pyogenes* for 6 h, the cells were washed with EGM-2. CFSE-labeled THP-1 cells (4 × 10^5^ cells/ml) were then layered over the HUVEC monolayers and incubated for 24 h at 37°C in 5% CO_2_. The cells were washed with EGM-2 and fixed in 1% glutaraldehyde in PBS. The fluorescent intensity of CFSE-labeled THP-1 cells was analyzed by fluorescent microscopy (BIOREVO BZ-9000; Keyence, Osaka, Japan) and the associated analysis software package (BZ-H2A).

### Cytotoxicity assay

HUVECs (confluent) and THP-1 cells (4 × 10^5^ cells/ml) were incubated with SlaA and C134A at 37°C for 24 h. Viable cells were determined using AlamarBlue cell-viability reagent (Bio-Rad, Kidlington, UK), in accordance with the manufacturer's instructions.

### Quantitative real-time RNA

Gene expression in HUVECs and the aorta was quantified using quantitative real-time PCR. Briefly, RNA was extracted from cell lysates using TRI Reagent (Molecular Research Center) and quantified by spectrometry at 260 and 280 nm. The RNA was reverse-transcribed using SuperScript VILO Master Mix (Thermo Fisher Scientific, Carlsbad, CA, USA), and quantitative real-time PCR with cDNA was performed with the StepOnePlus Real-time PCR system (Thermo Fisher Scientific), in accordance with the manufacturer's protocol. TaqMan probes, sense primers, and antisense primers for the expression of a housekeeping gene (*GAPDH*), ICAM1 (*ICAM1*), VCAM1 (*VCAM1*), E-selectin (SELE), or P-selectin (SELP) mRNA were purchased from Thermo Fisher Scientific.

### Immunofluorescence analysis

rSlaA-treated HUVECs were fixed and permeabilized using a cell fixation and permeabilization kit (Thermo Fisher Scientific), in accordance with the manufacturer's instructions, followed by incubation of the cells in a blocking solution (Thermo Fisher Scientific) for 30 min. The samples were stained with PE anti-human ICAM1 antibody (Biolegend, San Diego, CA, USA) or PE anti-human VCAM1 antibody (Biolegend) in the blocking solution. After overnight incubation at 4°C in the dark, the cells were washed. The intensity of the fluorescence of each sample was analyzed by fluorescent microscopy (BioRevo model BZ-9000; Keyence) and the associated analysis software package (BZ-H2A).

### *In vivo* analysis

Balb/c mice (five mice each) were injected i.v. with 30 μg/kg SlaA or PBS every 3 days for the indicated periods. Aspirin (100 mg/kg) and its vehicle (0.5% CMC) were administered i.p. 3 h prior to the injection of SlaA. The treated mice were sacrificed at several time points after this administration, after which the aorta was extirpated.

### Statistical analysis

Data were analyzed using GraphPad Prism 6.05 (GraphPad Software, La Jolla, CA, USA). All results are expressed as the mean ± SEM. The group means were compared using one-way analysis of variance. *P*-values of 0.05 or less were considered statistically significant.

## Results

### *S. pyogenes* secretes SlaA into the culture supernatant

Table 1 shows the association between the status of the *slaA* gene and the clinical manifestations associated with *S. pyogenes*. In PCR analyses, the *slaA* gene was detected among M3 and M4 serotypes of *S. pyogenes*, but not in the M1, M11, M12, M28, M75, and M89 serotypes, under the experimental conditions applied. A previous study showed that *S. pyogenes* encoding the *slaA* gene significantly upregulated SlaA production when cultured on D562 human pharyngeal epithelial cells (Sitkiewicz et al., 2006). Therefore, we tested the release of the SlaA protein from 27 strains of *S. pyogenes* co-cultured with HUVECs. By western blot analyses, we found that the SlaA protein was only detected in culture supernatant from *S. pyogenes* encoding the *slaA* gene (Table 1).

### Monocyte adhesion to endothelial cells

**Figure 1:**
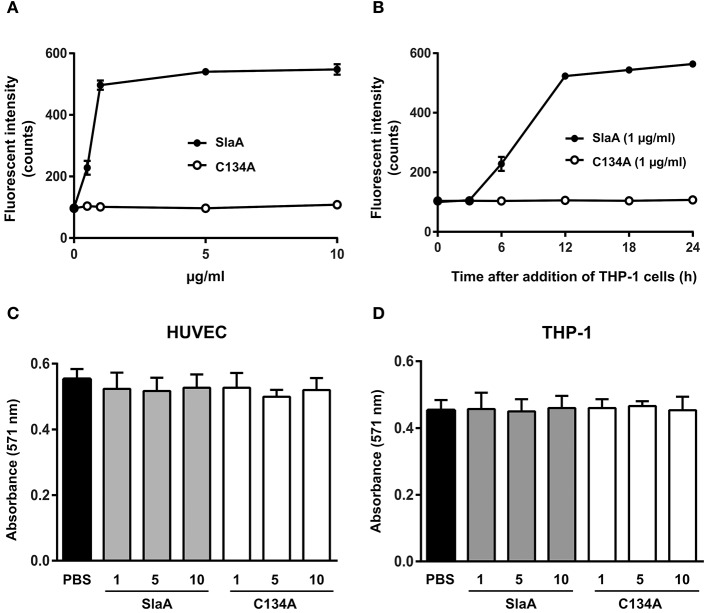
SlaA stimulated the adhesion of THP-1 cells to HUVECs. **(A)** HUVECs were incubated with various concentrations **(A)** or 1 μg/ml **(B)** of SlaA or C134A at 37°C for 6 h. The cells were incubated with CFSE-labeled THP-1 cells at 37°C for 24 h **(A)** or the indicated times **(B)**. These cells were washed and the labeled THP-1 cells were viewed with a fluorescent microscope. The fluorescent intensity of CFSE-labeled THP-1 cells was quantified as described in Materials and Methods. **(C,D)** HUVECs **(C)** and THP-1 cells **(D)** were incubated with various concentrations of SlaA and C134A at 37°C for 24 h, and then stained with AlamarBlue. The results shown represent the mean ± SEM; *n* = 4.

To investigate whether SlaA enhances the adhesion between HUVEC and THP-1 cells, we incubated SlaA-stimulated HUVEC with THP-1 cells. SlaA dose-dependently enhanced the adhesion of these two types of cells (Figure 1A). The interaction level peaked within 12 h and then remained at the same level (Figure 1B). The C134A variant, which does not have phospholipase A2 activity (Supplementary Figure 1), did not induce the adhesion of THP-1 cells to HUVECs (Figures 1A,B), showing that the enzymatic activity of SlaA is essential for this event.

To determine whether SlaA caused cytotoxicity, SlaA and C134A were incubated with HUVECs or THP-1 cells at 37°C for 24 h, and each type of treated cell was stained with AlamarBlue. SlaA and C134A did not have a detrimental effect on the growth, cell morphology, and membrane integrity (Figures 1C,D).

### *S. pyogenes* strain SSI-1 culture supernatant enhances the interaction between HUVECs and THP-1 cells

**Figure 2:**
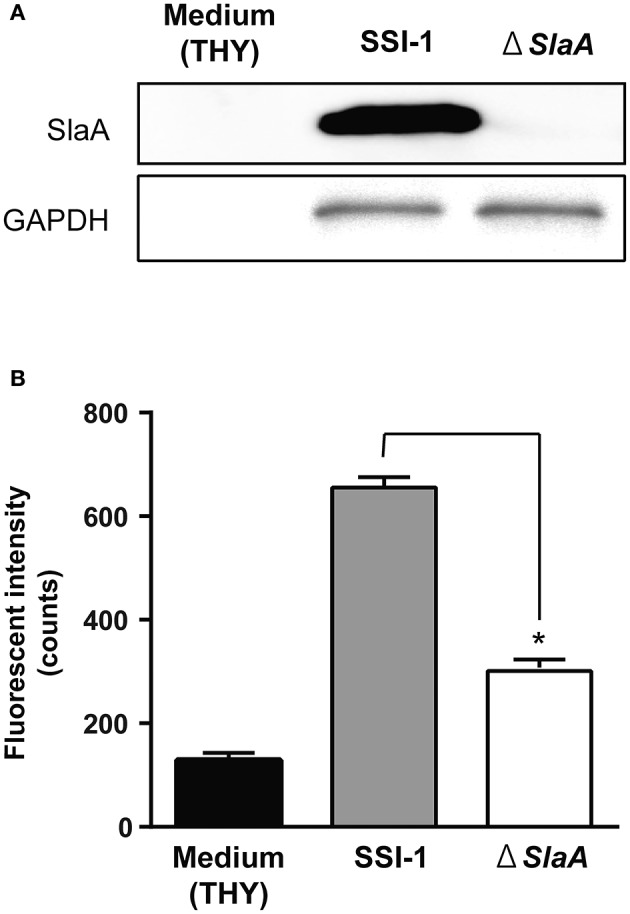
*Streptococcous pyogenes* culture supernatant induces the adhesion of THP-1 cells to HUVECs. **(A)** Protein expression of SlaA and GAPDH/Plr from *S. pyogenes* wild-type strain SSI-I and its Δ*SlaA* isogenic mutant strain was detected by Western blotting. **(B)** HUVECs were pre-incubated with THY broth, *S. pyogenes* SSI-I culture supernatant, or its Δ*SlaA* mutant strain culture supernatant at 37°C for 6 h. The cells were incubated with CFSE-labeled THP-1 cells at 37°C for 24 h. These cells were washed and the CFSE-labeled THP-1 cells were viewed with a fluorescent microscope. The fluorescent intensity of CFSE-labeled cells was quantified as described in Materials and Methods. The results shown represent the mean ± SEM; *n* = 5. Data were analyzed using a one-way ANOVA with Dunnett's multiple comparison test. Significant differences from the *S. pyogenes* SSI-I culture supernatant group are shown: ^*^P < 0.01.

We next confirmed the effect of *S. pyogenes* strain SSI-1 cultured supernatant on the adhesion of THP-1 cells to HUVECs. The phospholipase A2 enzymatic activity of culture supernatant of Δ*slaA* isogenic mutant was not detected (Supplementary Figure 1). *S. pyogenes* wild-type strain on a HUVEC monolayer expressed SlaA in culture supernatant, but Δ*slaA* isogenic mutant strain did not (Figure 2A). The culture supernatant of the wild-type strain enhanced the adhesion of THP-1 cells to HUVECs, but that of Δ*slaA* isogenic mutant had a limited effect under our experimental conditions (Figure 2B).

### SlaA induced the expression of adhesion molecules in HUVECs

**Figure 3:**
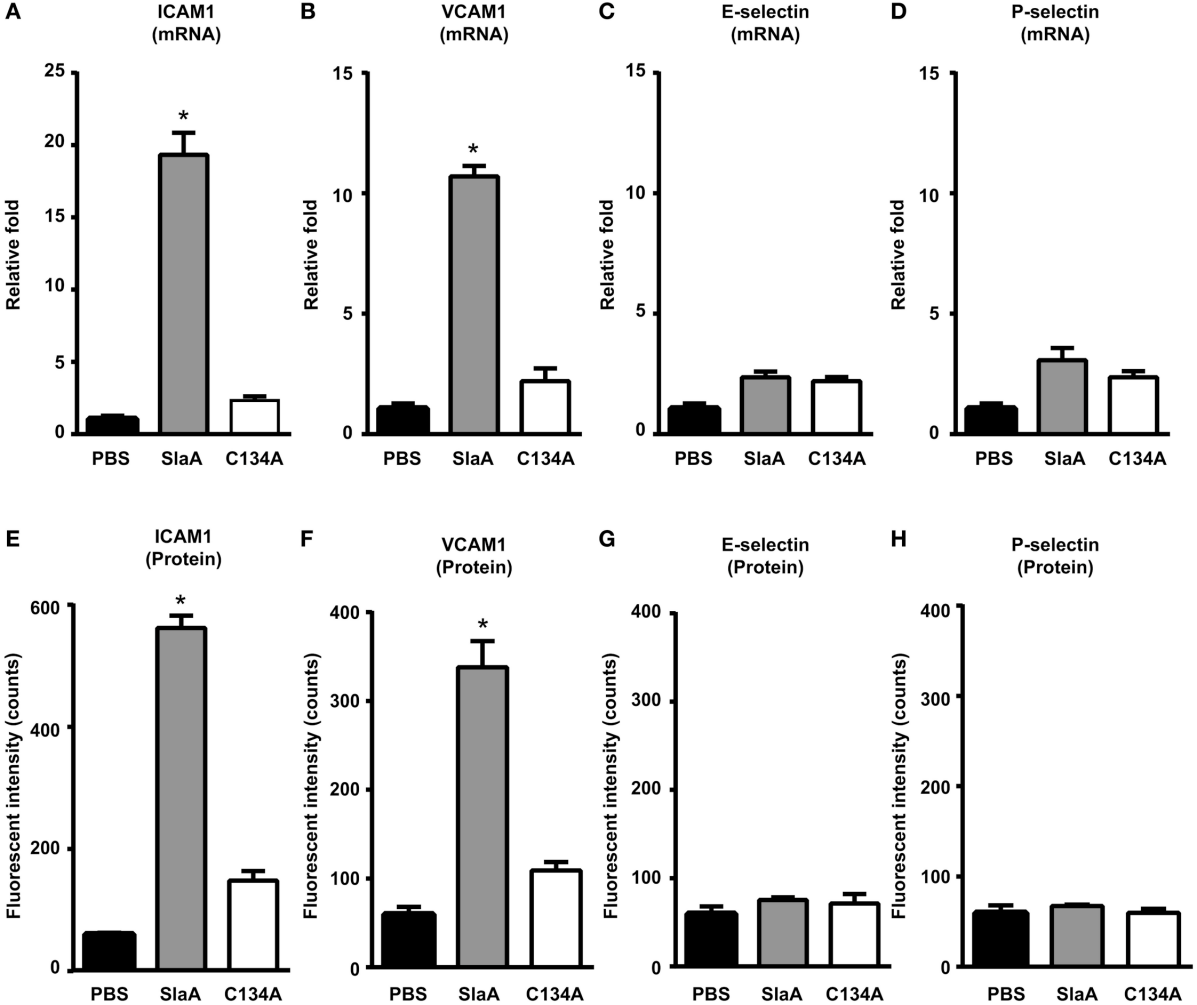
SlaA induced the expression of adhesion molecules in HUVECs. HUVECs were incubated with 1 μg/ml SlaA or C134A at 37°C for 3 h **(A–D)** and 6 h **(E–H)**. The transcription of ICAM1 **(A)**, VCAM1 **(B)**, E-selectin **(C)**, and P-selectin **(D)** mRNAs in HUVECs was measured by real-time PCR, as described in Materials and Methods. The relative quantity of these mRNAs was normalized to the relative quantity of GAPDH mRNA. The protein expression of ICAM1 **(E)**, VCAM1 **(F)**, E-selectin **(G)**, and P-selectin **(H)** in HUVECs was analyzed by immunostaining using fluorescently labeled antibodies. The fluorescence intensity of each protein was quantified as described in Materials and Methods. The results shown represent the mean ± SEM; *n* = 5. Data were analyzed using one-way ANOVA with Dunnett's multiple comparison test. Significant differences from the PBS group are shown: ^*^P < 0.01.

To investigate whether SlaA induced the expression of adhesion molecules in HUVECs, HUVECs were incubated with SlaA. SlaA upregulated the transcription of ICAM1 and VCAM1 mRNAs and the expression of ICAM1 and VCAM1 proteins, but C134A did not (Figures 3A,B,E,F). However, SlaA did not upregulate the transcription of E-selectin and P-selectin mRNAs and the expression of their proteins (Figures 3C,D,G,H).

### Aspirin inhibited the expression of adhesion molecules

**Figure 4:**
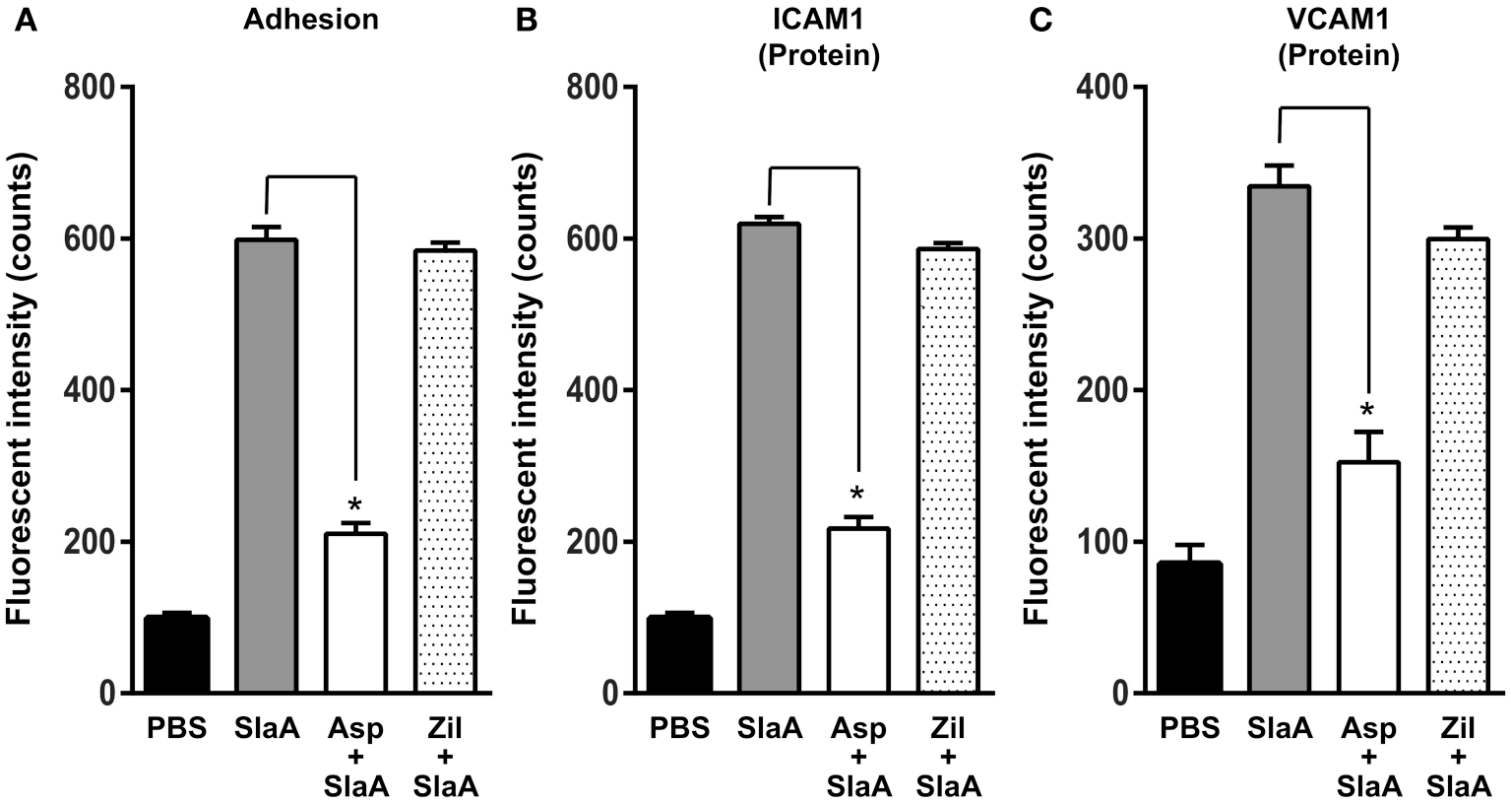
Aspirin inhibited the expression of adhesion molecules. HUVECs were pre-incubated with 5 mM aspirin and 50 μM zileuton at 37°C for 60 min, and then the cells were incubated with SlaA at 37°C for 6 h. **(A)** The treated cells were incubated with CFSE-labeled THP-1 cells at 37°C for 24 h. Adhesion of THP-1 cells to HUVECs **(A)** and the expression of ICAM1 **(B)** and VCAM1 **(C)** on the cells were analyzed as described in Materials and Methods. The results shown represent the mean ± SEM; *n* = 4. Data were analyzed using one-way ANOVA with Dunnett's multiple comparison test. Significant differences from the SlaA-treated group are shown: ^*^P < 0.01.

Phospholipase A2 is a key enzyme in the arachidonic acid cascade. We investigated the effect of arachidonic acid cascade inhibitors such as aspirin (cyclooxygenase 2 inhibitor) and zileuton (5-lipoxygenase inhibitor) on the adhesion of THP-1 cells to HUVECs and the expression of ICAM1 or VCAM1. HUVECs were treated with 5 mM aspirin or 50 μM zileuton at 37°C for 60 min. Aspirin inhibited the adhesion of THP-1 cells to HUVECs and the expression of ICAM1 or VCAM1, but zileuton did not (Figure 4).

### SlaA upregulated the transcription of ICAM1 and VCAM1 mRNAs in mouse Aorta

**Figure 5:**
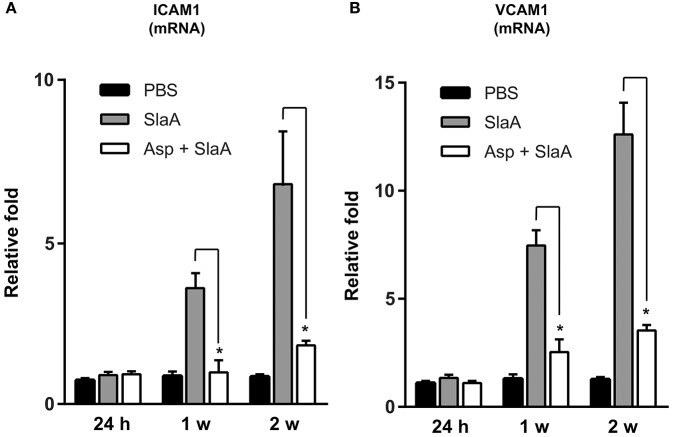
SlaA upregulated the transcription of ICAM1 and VCAM1 mRNAs in mouse aorta. Balb/c mice (five mice each) were injected i.v. with 30 μg/kg SlaA or PBS every 3 days for the indicated periods. Aspirin (100 mg/kg) and its vehicle (0.5% CMC) were administered i.p. 3 h prior to the injection of SlaA. The transcription of ICAM1 **(A)** and VCAM1 **(B)** mRNAs in the vascular endothelium of aorta was measured by real-time PCR as described in Materials and Methods. The relative quantities of these mRNAs were normalized to the level of GAPDH mRNA. Data were analyzed using one-way ANOVA. Significant differences from the SlaA-treated group are shown: ^*^P < 0.01.

We investigated whether SlaA stimulated the transcription of ICAM1 and VCAM1 mRNAs in mouse aorta. The mice were intravenously administered 30 μg/kg SlaA every 3 days. SlaA upregulated the transcription of ICAM1 and VCAM1 mRNAs at the aorta at 1 and 2 weeks after the initial injection, but did not at 24 h. The upregulation of these mRNAs in the aorta of mice injected with SlaA was inhibited by i.p. administration of 100 mg/kg aspirin (Figures 5A,B).

## Discussion

As shown in Table 1, SlaA is known to be mainly released from M3 strains of *S. pyogenes*, and is encoded on a prophage genome. The serotype M3 GAS strains of *S. pyogenes* expressing SlaA and some M3 GAS strains have been shown to be particularly invasive (Beres et al., 2002, 2004; Banks et al., 2004; Nagiec et al., 2004). We also detected the *slaA* gene in the SSI-1 strain, which is a serotype M3 strain derived from a patient with an invasive infection (Terao et al., 2002), and the SlaA protein in the culture supernatant of *S. pyogenes* co-cultured with HUVECs. Recently, there has been an increase in infectious diseases induced by *S. pyogenes* carrying the *slaA* gene. Previous reports described that SlaA contributed to the colonization and infection of *S. pyogenes* in pharyngeal epithelial cells (Sitkiewicz et al., 2006, 2007). In addition, Sitkiewicz et al. (2006) reported that the Δ*slaA* isogenic mutant strain caused significantly less morbidity than the wild-type strain. However, the pathogenesis of SlaA is still not completely understood. In the present study, we have shown that SlaA from *S. pyogenes* induced the expression of ICAM1 and VCAM1 in HUVECs, resulting in the enhanced adhesion of THP-1 cells to HUVECs. In addition, SlaA also stimulated the expression of ICAM1 and VCAM1 in mouse aorta, and the pre-administration of aspirin inhibited these events.

As cytosolic and secretory phospholipase A2 in mammalian cells is known to be related to the expression of ICAM1 (Thommesen et al., 1998; Zhu et al., 1999; Barnett et al., 2001; Yu et al., 2012), we examined whether SlaA induced the expression of adhesion molecules such as ICAM1 in HUVECs. SlaA upregulated the transcription of ICAM1 and VCAM1 mRNAs and the expression of ICAM1 and VCAM1 proteins, resulting in the adhesion of THP-1 cells. On the other hand, C134A did not stimulate these events, suggesting that the PLA2 activity of SlaA is essential for them. The expression of E-selectin and P-selectin was not detected under our experimental conditions. In addition, aspirin inhibited the expression of ICAM1 and VCAM1 in HUVECs treated with SlaA, but zileuton did not. This suggested that the arachidonic acid cascade via cyclooxygenase 2 plays an important role in the expression of adhesion molecules in HUVECs treated with SlaA. The culture supernatant of Δ*slaA* isogenic mutant strain did not result in complete absence of the adhesion of THP-1 cells to HUVECs. This suggests that other secretory proteins besides SlaA participate in the adhesion of monocytes to endothelial cells.

Consequently, we suggested that the SlaA-induced expression of ICAM-1 and VCAM-1 may contribute to the leukostasis, vascular injury, and capillary leakage characteristics of invading *S. pyogenes* infection. Further studies are needed to clarify the mechanism behind the inflammation of endothelial cells treated with SlaA.

## Author contributions

Conceived and designed the experiments: MO and YT. Performed the experiments: MO, HD, MK, and TI. Preparation of the materials: MY and SK. Analyzed the data: MO, TI, and TM. Wrote the paper: MO and YT.

### Conflict of interest statement

The authors declare that the research was conducted in the absence of any commercial or financial relationships that could be construed as a potential conflict of interest.
