# Supplementary material for: Streptococcus pyogenes Phospholipase A2 Induces the Expression of Adhesion Molecules on Human Umbilical Vein Endothelial Cells and Aorta of Mice
Source: Front Cell Infect Microbiol. 2017 Jun 30;7:300. doi: 10.3389/fcimb.2017.00300 (PMC5491884; doi:10.3389/fcimb.2017.00300)
Supplement: Supplementary file 1 [file DataSheet1.docx]

**Supplementary figure 1**





**Enzymatic activity of recombinant protein and culture supernatants.**

The enzymatic activity of rSlaA, C134A, and the culture supernatant of SSI-1 and Δ*slaA* was measured by phospholipase A2 assay kit. Data were analyzed using one-way ANOVA with Dunnett’s multiple comparison test. Significant differences from the PBS and THY medium group are shown: **P* < 0.01.
